# Phase-specific turbulence index derived from vector flow imaging for identifying intraplaque neovascularization in carotid plaques

**DOI:** 10.3389/fcvm.2026.1831573

**Published:** 2026-06-17

**Authors:** Qian Wang, Kefangyuan Zheng, Donghua Xian, Liuping Cui, Jiarui Bao, Yalan Fang

**Affiliations:** 1 Department of Neurology, The Second Hospital of Shanxi Medical University, Taiyuan, Shanxi, China; 2 Second Clinical College, Shanxi Medical University, Taiyuan, Shanxi, China

**Keywords:** atherosclerosis, hemodynamics, intraplaque neovascularization, turbulence index, vector flow imaging

## Abstract

**Objectives:**

To investigate the association between phase-specific turbulence index (Tur) derived from vector flow imaging (VFI) and intraplaque neovascularization (IPN) in carotid atherosclerotic plaques, and to evaluate its potential value as a noninvasive hemodynamic biomarker for IPN assessment.

**Methods:**

In this prospective study, 108 carotid plaques were analyzed. Tur was measured using VFI at peak systole and end-diastole at three locations relative to the plaque: upstream, the thickest region, and downstream. Intraplaque neovascularization was assessed by superb microvascular imaging (SMI) and classified as negative or positive. Least absolute shrinkage and selection operator (LASSO) regression was used for exploratory predictor selection, and an exploratory predictive model was internally validated using receiver operating characteristic analysis, calibration curves, and bootstrap resampling.

**Results:**

Among the 108 plaques, 82 (75.9%) were IPN-positive and 26 (24.1%) were IPN-negative. The downstream Tur during end-diastole was significantly lower in IPN-positive plaques compared with IPN-negative plaques (*P* = 0.008). An exploratory multivariable model incorporating diastolic blood pressure, downstream end-diastolic Tur, plaque length, plaque thickness, female sex, and plaque location showed acceptable internal discrimination, with an area under the receiver operating characteristic curve (AUC) of 0.830, and acceptable calibration. After bootstrap correction, the bias-corrected C-statistic was 0.768.

**Conclusion:**

Reduced downstream end-diastolic Tur was associated with IPN in carotid plaques. Phase-specific Tur derived from VFI may provide a potential noninvasive hemodynamic marker for IPN assessment.

## 1 Introduction

Ischemic stroke (IS) is the second leading cause of death worldwide, with carotid atherosclerotic stenosis representing a major contributing etiology (1). Rupture of vulnerable atherosclerotic plaques and subsequent thromboembolism are critical events leading to IS (2). Intraplaque neovascularization (IPN) promotes plaque instability and is recognized as an independent risk factor for IS (3, 4). Hemodynamic forces play a pivotal role in the initiation and progression of carotid atherosclerosis, influencing vascular homeostasis, tone, and endothelial integrity.

Vector flow imaging (VFI) is a recently developed, angle-independent ultrasound technique that enables simultaneous quantification of both the magnitude and direction of blood flow velocity (5). It allows accurate measurement of wall shear stress (WSS), the tangential force exerted by blood flow on the vessel wall. Altered WSS has been associated with plaque progression, instability and increasing the risk of plaque rupture (6–8). VFI also provides the turbulence index (Tur), which quantifies the directional dispersion of blood flow, reflecting local flow disturbance. Tur values range from 0 to 1, or from 0% to 100%, with higher values indicating greater flow disturbance (9–11). Importantly, blood flow in arteries exhibits significant temporal variation during the cardiac cycle, and recent studies suggest that phase-specific hemodynamic patterns may have distinct physiological and pathological implications (9, 12). However, whether cardiac phase–dependent turbulence characteristics are associated with IPN remains largely unexplored.

Despite the established role of IPN in plaque vulnerability, current noninvasive assessment methods have limitations (2, 4). Contrast-enhanced ultrasound (CEUS) is commonly used for IPN assessment but requires intravenous contrast agents, which may limit its routine applicability (13, 14). Superb microvascular imaging (SMI) offers a contrast-free alternative for plaque neovascularization assessment, although it remains semi-quantitative and operator-dependent (13–15). However, CEUS and SMI mainly characterize microvascular signals within the plaque and do not directly describe the phase-specific local hemodynamic environment around the lesion. Therefore, whether phase-specific Tur derived from VFI is associated with IPN and whether it could serve as an imaging biomarker for plaque vulnerability remain unknown.

Therefore, the aim of this study was to investigate the relationship between phase-specific turbulence parameters derived from VFI and intraplaque neovascularization in carotid atherosclerotic plaques. In addition, we evaluated whether these turbulence-derived hemodynamic signatures could serve as potential imaging biomarkers for identifying plaques with IPN. By integrating advanced hemodynamic assessment with plaque imaging, this study seeks to provide new insights into the hemodynamic mechanisms underlying plaque vulnerability and to explore the potential clinical utility of VFI-derived parameters in risk stratification of carotid atherosclerosis.

## 2 Methods

### 2.1 Study design

This single-center, prospective, observational cross-sectional study was approved by the institutional ethics committee (Approval No. [2025]YX No. 384). All participants provided written informed consent.

### 2.2 Study population

**Figure 1:**
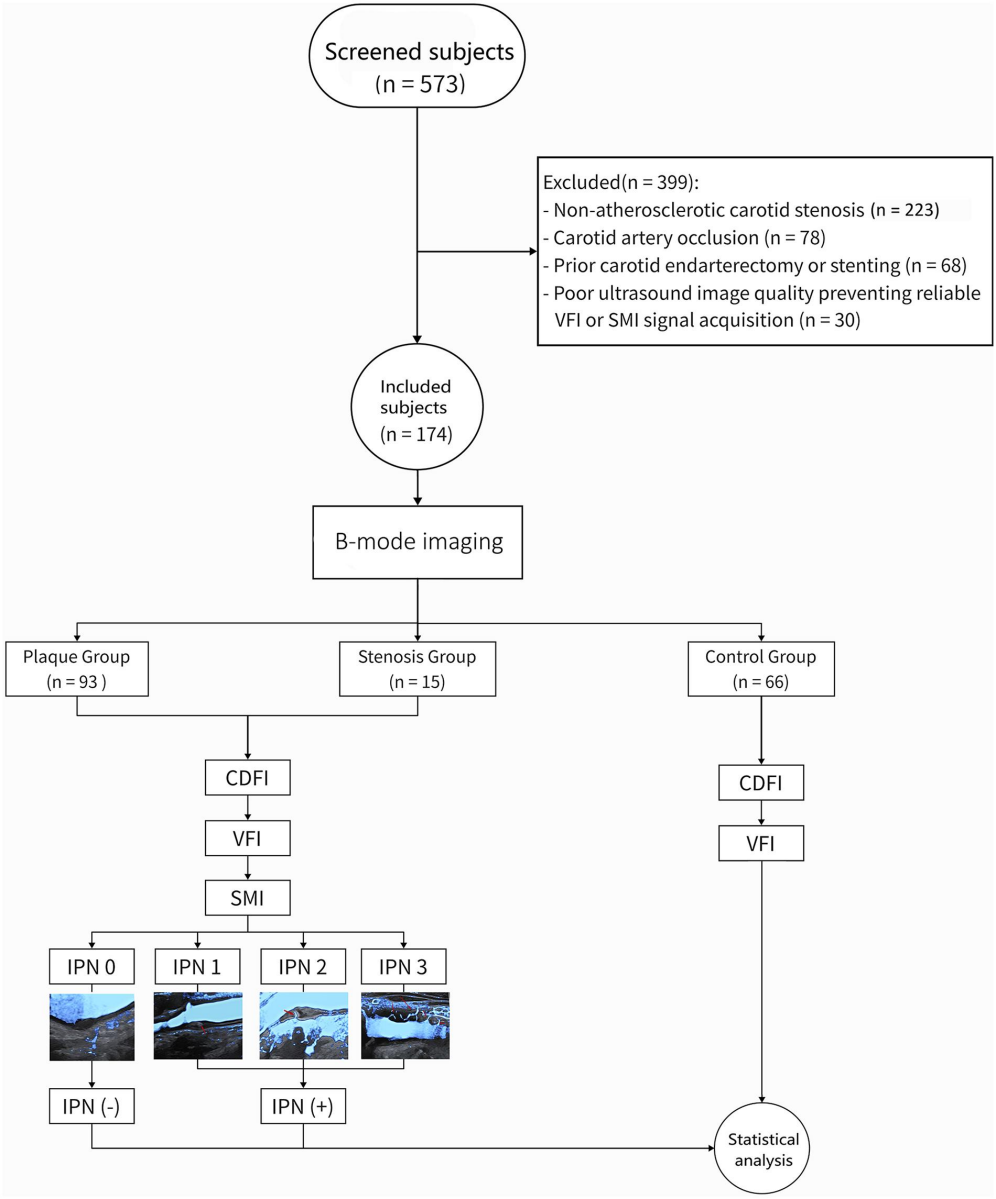
Study flowchart and IPN classification workflow. A total of 573 subjects were screened, and 174 subjects were included after exclusion. Participants were classified into plaque, stenosis, and control groups. Plaques were further evaluated using color Doppler flow imaging (CDFI), VFI, and SMI. SMI grade 0 was defined as IPN-negative, whereas grades 1–3 were defined as IPN-positive.

A total of 573 consecutive subjects were recruited between November 2025 and January 2026. After applying inclusion and exclusion criteria, 174 subjects were included in the final analysis (Figure 1). Inclusion criteria were: (1) age ≥18 years; (2) carotid ultrasound findings consistent with normal, plaque, or stenosis definitions; and (3) provision of informed consent. Exclusion criteria were: (1) non-atherosclerotic carotid stenosis; (2) carotid occlusion; (3) prior carotid endarterectomy or stenting; (4) poor image quality precluding VFI or SMI analysis.

Plaque was defined as intima-media thickness ≥1.5 mm, according to the American Society of Echocardiography (16). Carotid stenosis (≥50% luminal diameter reduction) was defined based on the European Carotid Surgery Trial criteria (17). Subjects were categorized into three groups: control (bilateral intima-media thickness <1.0 mm, no plaque); plaque (at least one plaque with stenosis <50%); and stenosis (luminal stenosis ≥50% confirmed by digital subtraction angiography, computed tomography angiography, or magnetic resonance angiography). For participants with multiple eligible plaques, the target plaque was first selected from lesions that could be completely visualized in the same longitudinal plane and met the image-quality requirements for both VFI and SMI analysis. If more than one plaque met these imaging criteria, the plaque causing the most severe stenosis was selected for analysis.

### 2.3 Clinical data collection

Demographic and clinical data, including age, sex, height, weight, medical history, and laboratory results, were recorded. Systolic and diastolic blood pressure and heart rate were measured at rest.

### 2.4 Ultrasound examination

All examinations were performed using a Mindray Resona A20 ultrasound system (Shenzhen Mindray Bio-Medical Electronics Co., Ltd.) equipped with an LM18-5WU matrix linear array probe (frequency range, 3.8–18 MHz). Subjects were examined in the supine position with the neck slightly extended. Scans were performed by a physician with >5 years of experience in carotid ultrasound.

### 2.5 B-Mode and color Doppler flow imaging

Longitudinal scans were obtained from the common carotid artery origin to the extracranial internal carotid artery to assess intima-media thickness, plaque location, morphology, and dimensions by B-mode imaging. Percentage diameter stenosis was calculated using the European Carotid Surgery Trial method. Peak systolic velocity (PSV) and end-diastolic velocity (EDV) were measured using color Doppler flow imaging (CDFI).

### 2.6 VFI measurement of Tur

In B-mode imaging, the target plaque was first identified, and the probe was then adjusted to fully display the plaque in the longitudinal plane before VFI was activated. For VFI image acquisition, a standardized acquisition region of interest (ROI) was used, with a size of 3 cm × 2 cm, imaging depth of <4 cm, arrow density of 10%, and velocity scale of 30 cm/s.

**Figure 2:**
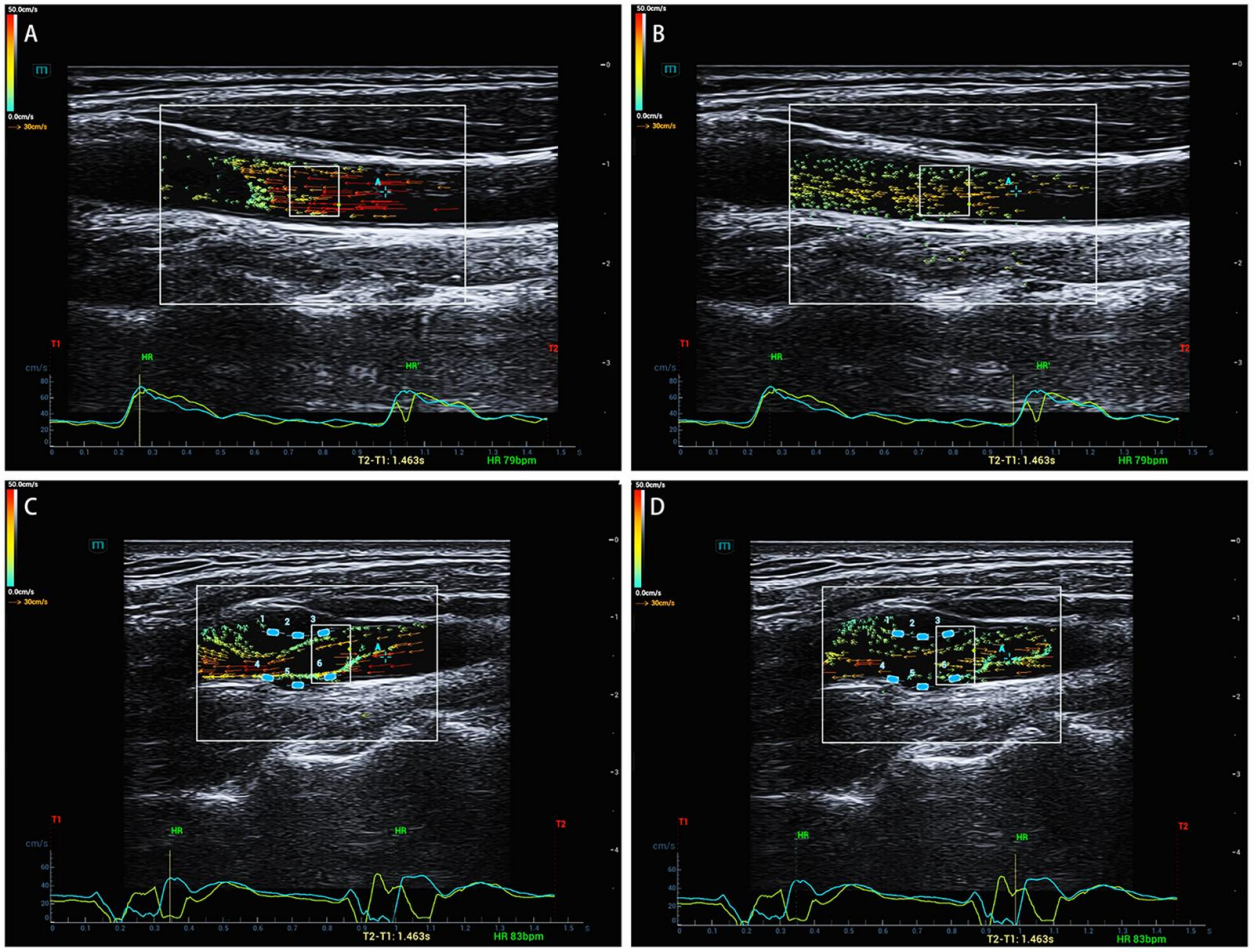
Tur measurement process. Arrow colors transition from green to red representing blood flow velocity, with a scale of 30 cm/s. Point A is positioned at the proximal end to capture the cardiac cycle, indicated by the blue HR-HR' curve. The ROI corresponds to the Tur measurement box, with the light green curve depicting blood flow velocity. **(A,B)** show carotid Tur measurements in the healthy group, with A representing systole and B representing end-diastole. C and D show Tur measurements in the plaque group. Points 1–3 mark the midpoint downstream of the plaque, the thickest point, and the midpoint upstream of the plaque, respectively. Points 4–6 are marked at the corresponding locations along the opposite-side vessel wall. The ROI measurement box is placed to obtain corresponding cardiac cycle Tur results. **(C)** represents systole, and **(D)** represents end-diastole.

The VFI system automatically calculated Tur values from multiple cardiac cycles during image acquisition and provided averaged results. Stored cine loops were required to include at least one complete cardiac cycle to allow accurate offline identification of systolic and diastolic phases. Tur was measured on the stored cine loops using a separate measurement ROI. This measurement ROI was placed to cover the local flow field along the plaque-side and opposite-side vessel walls. In the control group, the center of the measurement ROI was placed in the mid-lumen of the middle common carotid artery, and its size was adjusted according to vessel diameter, usually approximately 0.5 cm × 0.5 cm. In the plaque group, the centers of the measurement ROIs were placed upstream of the plaque, the thickest plaque site, and downstream of the plaque, respectively. The size of each Tur measurement ROI was adjusted according to vessel diameter and plaque length. After ROI placement, the system automatically generated the total average turbulence index (TATur) for each ROI. Peak systole and end-diastole were then identified by sliding the time axis to the maximal and minimal flow velocity points, respectively, then peak systolic Tur and end-diastolic Tur were recorded (Figure 2).

### 2.7 SMI assessment of IPN

Dynamic SMI scanning of the plaque was performed in the longitudinal plane for 3 min, and videos were recorded. IPN grading was performed independently by two experienced physicians blinded to clinical and imaging data. Grading criteria were as follows (13, 15): Grade 0, no blood flow signals within the plaque; Grade 1, few dot-like or short linear signals; Grade 2, moderate signals; Grade 3, abundant, reticular, or branching signals.

### 2.8 Statistical analysis

Statistical analyses were performed using R version 4.5.2 and GraphPad Prism version 10.1.2. Continuous variables were expressed as mean ± standard deviation or median (interquartile range), as appropriate. Categorical variables were expressed as frequencies (percentages). Group comparisons were performed using the Kruskal–Wallis test, one-way ANOVA, chi-square test, or Fisher's exact test, as appropriate. *Post hoc* pairwise comparisons for non-parametric data were performed using Dunn's test with Bonferroni correction.

Because of the limited sample size, the prediction model was developed as an exploratory model. Candidate predictors were first screened using univariate logistic regression, and variables with *P* < 0.10 were entered into least absolute shrinkage and selection operator (LASSO) regression with 10-fold cross-validation. The *λ*.1se solution was used to obtain a more parsimonious model. Model discrimination was assessed using the area under the receiver operating characteristic curve (AUC). Calibration was evaluated using the Hosmer–Lemeshow test and Brier score. Internal validation was performed using 1,000 bootstrap resamples to estimate optimism and bias-corrected C-statistics. Inter-rater reliability for IPN grading was assessed using Cohen's kappa coefficient.

## 3 Results

### 3.1 Baseline characteristics

A total of 174 subjects were included: 93 (53.4%) in the plaque group, 15 (8.6%) in the stenosis group, and 66 (37.9%) in the control group. Baseline characteristics are summarized in Table 1. No significant differences were observed among groups in hypertension, body mass index, blood pressure, glycated hemoglobin, homocysteine, triglycerides, HDL cholesterol, or total cholesterol/HDL ratio (all *P* > 0.05). Significant differences were observed in age, sex, diabetes, smoking history, total cholesterol, HDL, and LDL (all *P* *<* 0.05).

**Table 1:** Baseline characteristics.

Variables	All patients (*n* = 174)	Control group (*n* = 66)	Plaque group (*n* = 93)	Stenosis group (*n* = 15)	*p*
Sex					<**0.001**
Male	108 (62)	30 (45)	65 (70)	13 (87)	
Female	66 (38)	36 (55)	28 (30)	2 (13)	
Age, years	62.00 (55.00–69.00)	57.00 (50.00–62.00)	66.00 (59.00–70.00)	68.00 (59.00–74.00)	<**0.001**
Diabetes	39 (22)	8 (12)	29 (31)	2 (13)	**0** **.** **011**
Hypertension	87 (50)	28 (42)	53 (57)	6 (40)	0.140
Smoke exposure	71 (41)	16 (24)	47 (51)	8 (53)	**0** **.** **002**
BMI, kg/m^2^	24.49 (22.59–26.12)	24.71 (23.42–26.12)	24.34 (22.49–25.95)	24.49 (20.40–26.33)	0.430
SBP, mmHg	137.40 (126.00–151.00)	137.40 (123.00–148.00)	142.00 (130.00–155.00)	135.00 (124.00–146.00)	0.220
DBP, mmHg	83.00 (75.00–90.00)	84.00 (77.00–87.00)	80.00 (75.00–91.00)	84.00 (76.00–90.00)	0.930
HbA1c, %	6.10 (5.60–6.62)	6.20 (5.60–6.21)	6.00 (5.70–6.90)	6.20 (5.70–6.80)	0.520
HCY, mmol/L	13.82 (10.90–18.04)	13.34 (10.75–14.96)	13.70 (10.74–18.98)	16.45 (13.32–19.78)	0.055
TC, mmol/L	4.40 (3.52–4.95)	4.61 (3.93–5.16)	4.19 (3.50–4.88)	4.19 (3.08–4.92)	**0** **.** **040**
TG, mmol/L	1.52 (1.02–2.13)	1.57 (1.13–2.13)	1.48 (1.00–2.96)	1.43 (1.04–2.10)	0.950
HDL, mmol/L	1.08 (0.95–1.26)	1.26 (1.08–1.28)	1.04 (0.91–1.22)	1.08 (0.75–1.35)	**<0.001**
LDL, mmol/L	2.37 (1.85–3.12)	2.92 (2.18–3.16)	2.26 (1.71–2.79)	2.19 (1.52–2.86)	**0** **.** **002**
HDL/CHOL	27.18 (22.22–30.83)	28.52 (23.22–31.23)	27.18 (22.08–30.83)	25.16 (20.27–30.28)	0.490
Location					**<0.001**
CCA	83 (48)	66 (100)	16 (17)	1 (7)	
CB	63 (36)		61 (66)	2 (13)	
ICA	28 (16)		16 (17)	12 (80)	
PSV, cm/s	73.59 (57.43–90.14)	75.38 (67.40–88.94)	62.62 (48.66–85.75)	141.60 (78.75–289.98)	**<0.001**
EDV, cm/s	21.34 (15.95–25.92)	23.13 (19.14–28.72)	17.95 (13.56–23.13)	49.46 (21.96–71.84)	**<0.001**
RI	0.70 (0.65–0.76)	0.70 (0.66–0.74)	0.71 (0.64–0.77)	0.67 (0.59–0.76)	0.630
Plaque size					
Thickness, mm	2.50 (1.90–3.00)		2.40 (1.90–2.90)	3.60 (2.80–4.50)	**<0.001**
Length, mm	12.70 (9.65–18.90)		12.10 (9.50–16.60)	21.30 (14.00–22.50)	**0** **.** **004**

BMI, body mass index; CB, carotid bifurcation; CCA, common carotid artery; HbA1c, hemoglobin A1c; HCY, homocysteine; HDL, high-density lipoprotein cholesterol; HDL/CHOL, HDL cholesterol to total cholesterol ratio; ICA, internal carotid artery; LDL, low-density lipoprotein cholesterol; PSV, peak systolic velocity; RI, resistance index; TC, total cholesterol; TG, triglycerides.

Data are presented as number (%) or median (interquartile range).

Boldface entries indicate statistical significance.

### 3.2 Comparison of Tur across groups

**Figure 3:**
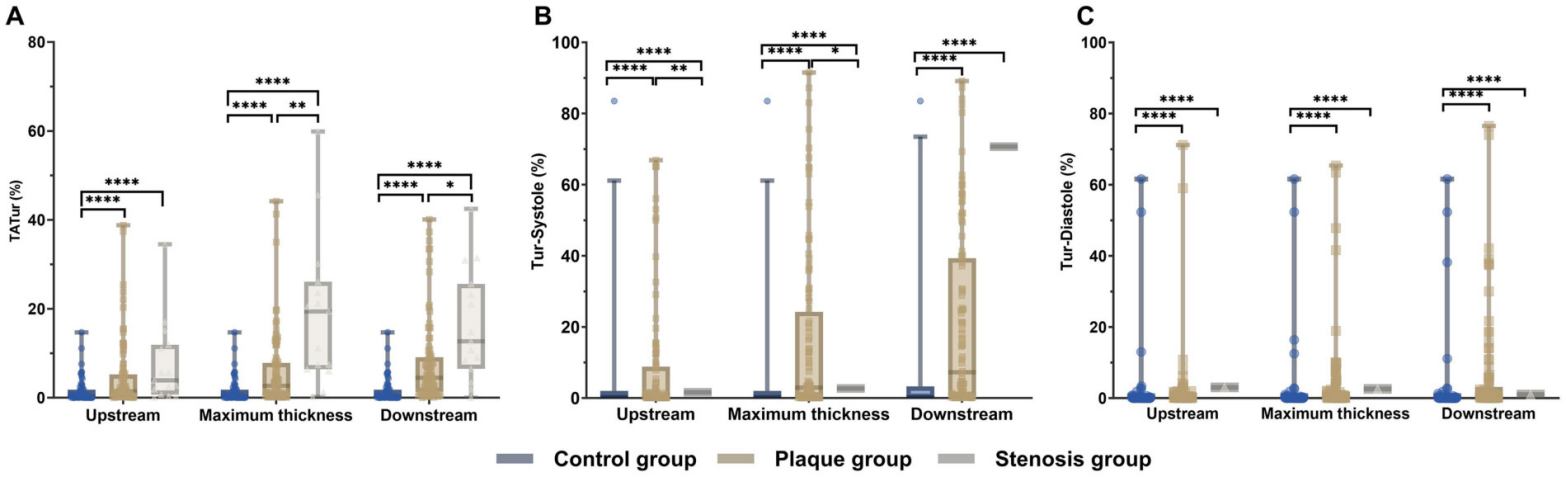
Comparison of Tur at three locations among the three groups in different phases of the cardiac cycle. **(A)** Comparison of total average turbulence index (TATur) at three locations among various groups. **(B)** Comparison of Tur-Systole at three locations among various groups. **(C)** Comparison of Tur-Diastole at three locations among various groups. *P < 0.05, **P < 0.01, ****P < 0.0001.

VFI successfully acquired Tur parameters in all subjects. At all measurement locations, TATur, Tur-Systole, and Tur-Diastole were significantly higher in the plaque and stenosis groups than in the control group (Kruskal–Wallis test, all adjusted *P* < 0.001). At the thickest part of the plaque, TATur (adjusted *P* < 0.01) and Tur-Systole (adjusted *P* *<* 0.05) were significantly higher in the stenosis group than in the plaque group. Downstream TATur was also significantly higher in the stenosis group (adjusted *P* *<* 0.05). No significant differences were found for upstream parameters or downstream Tur-Systole and Tur-Diastole between the plaque and stenosis groups (Table 2, Figure 3).

**Table 2:** Comparison of Tur among study groups at three locations in different phases of the cardiac cycle.

	TATur (%)			Tur-Systole (%)			Tur-Diastole (%)		
Group	Upstream	Maximum thickness	Downstream	Upstream	Maximum thickness	Downstream	Upstream	Maximum thickness	Downstream
Control group (*n* = 66)	0.20 (0.10–1.80)	0.20 (0.10–1.80)	0.20 (0.10–1.80)	0.10 (0.00–0.70)	0.10 (0.00–0.70)	0.10 (0.00–0.70)	0.10 (0.00–0.20)	0.10 (0.00–0.20)	0.10 (0.00–0.20)
Plaque group (*n* = 93)	1.40 (0.30–5.10)^a^	2.70 (0.50–7.59)^a^^,^^b^	4.50 (1.30–8.90)^a^^,^^b^	0.70 (0.10–5.00)^a^^,^^b^	2.00 (0.40–16.90)^a^^,^^b^	4.70 (0.50–29.90)^a^	0.30 (0.10–0.60)^a^	0.30 (0.10–1.20)^a^	0.50 (0.20–1.50)^a^
Stenosis group (*n* = 15)	3.90 (0.70–11.90)^c^	19.40 (6.40–26.10)^c^	12.70 (6.50–25.60)^c^	4.50 (0.80–39.70)^c^	55.00 (4.10–75.60)^c^	37.10 (11.60–55.50)^c^	0.30 (0.20–2.00)^c^	1.40 (0.30–7.50)^c^	1.00 (0.10–30.00)^c^
***p***	**<0.001**	**<0.001**	**<0.001**	**<0.001**	**<0.001**	**<0.001**	**<0.001**	**<0.001**	**<0.001**

Tur, turbulence index; TATur, total average turbulence index.

Boldface entries indicate statistical significance.

a Differences between the plaque group and the control group were statistically significant (*P* < 0.05).

b Differences between the plaque group and the stenosis group were statistically significant (*P* < 0.05).

c Differences between the stenosis group and the control group were statistically significant (*P* < 0.05).

### 3.3 Association between Tur and IPN

Of the 108 plaques assessed by SMI, 82 (75.9%) were IPN-positive (Grades 1–3) and 26 (24.1%) were IPN-negative (Grade 0). Tur-Diastole-Downstream was significantly lower in the IPN-positive group than in the IPN-negative group [median, 0.35% (IQR, 0.20–1.20) vs. 2.15% (IQR, 0.30–11.00); *P* *=* *0.008*]. No significant differences were observed for other Tur parameters (Table 3).

**Table 3:** Comparison of Tur in positive and negative states of IPN evaluated by SMI.

Variables	(+) (*n* = 82)	(−) (*n* = 26)	*p*
Upstream			
TATur (%)	1.50 (0.30–5.40)	2.70 (0.50–9.80)	0.420
Tur-Systole (%)	0.75 (0.20–5.40)	1.30 (0.40–11.20)	0.330
Tur-Diastole (%)	0.25 (0.10–0.50)	0.50 (0.10–2.80)	0.053
Maximum thickness			
TATur (%)	3.05 (0.60–8.60)	4.15 (0.60–13.80)	0.510
Tur-Systole (%)	2.00 (0.30–25.10)	9.55 (1.70–27.20)	0.100
Tur-Diastole (%)	0.30 (0.10–1.00)	1.40 (0.10–5.10)	0.110
Downstream			
TATur (%)	4.65 (1.30–9.30)	7.70 (2.30–13.40)	0.120
Tur-Systole (%)	5.60 (0.50–37.10)	17.40 (1.40–49.80)	0.200
Tur-Diastole (%)	0.35 (0.20–1.20)	2.15 (0.30–11.00)	**0** **.** **008**

Tur, turbulence index; TATur, total average turbulence index; IPN, intraplaque neovascularization; SMI, superb microvascular imaging.

Boldface entries indicate statistical significance.

### 3.4 Model development and validation

Univariate logistic regression identified eight candidate variables (*P* *<* 0.10): diastolic blood pressure, Tur-Diastole-Downstream, plaque length, plaque thickness, female sex, plaque location at the carotid bifurcation, systolic blood pressure, and Tur-Downstream. LASSO regression selected six variables with non-zero coefficients at the optimal *λ*.1se (0.0469): carotid bifurcation (coefficient, −0.254), female sex (–0.212), plaque thickness (0.122), plaque length (0.050), Tur-Diastole-Downstream (–0.020), and diastolic blood pressure (–0.016) (Supplementary Figure 1 and Table 1).

**Figure 4:**
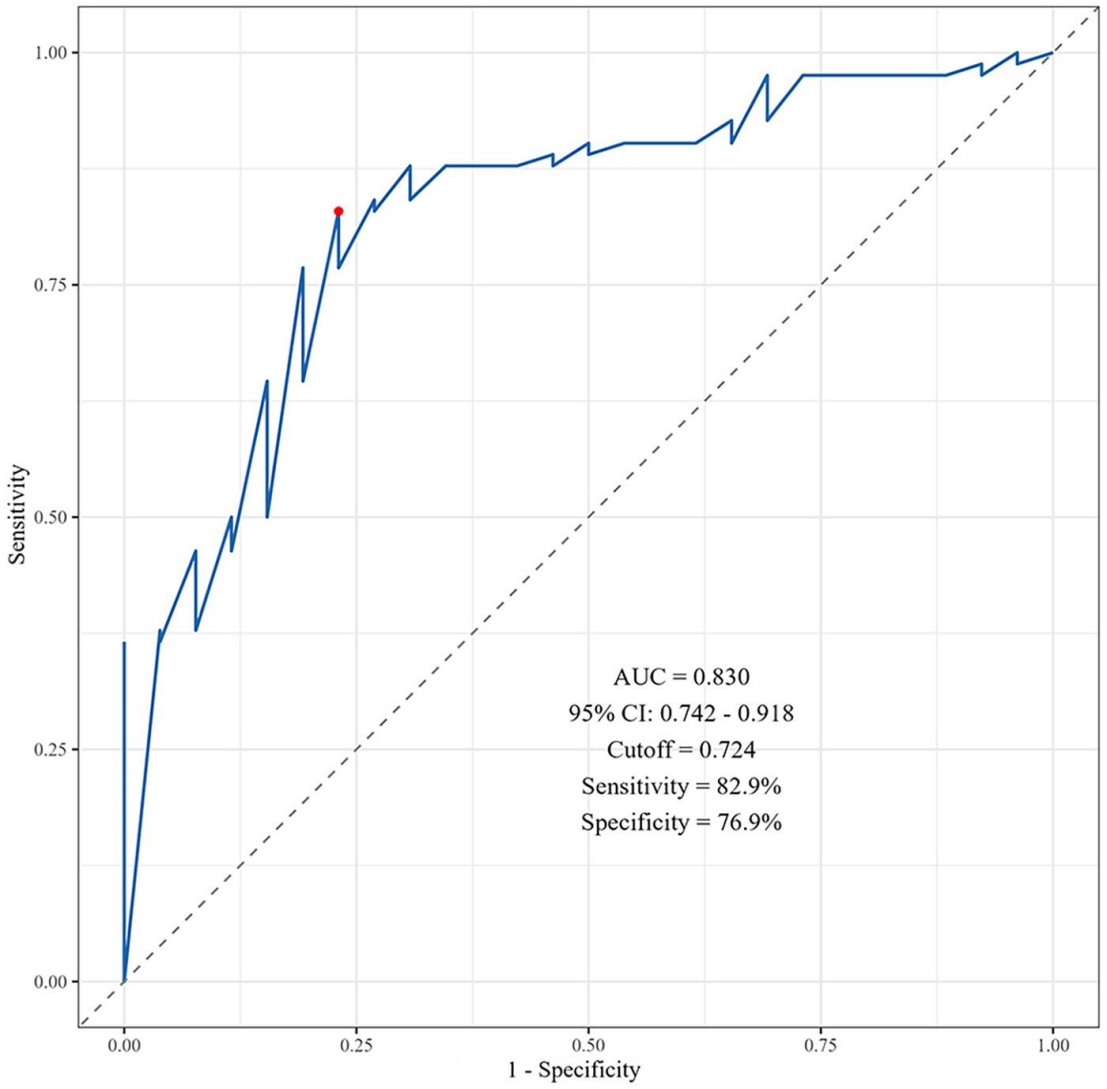
Evaluation of diagnostic performance for IPN using ROC curves. The ROC curve shows an AUC of 0.830, indicating acceptable diagnostic accuracy. The red dot represents the optimal cutoff value of 0.724, yielding a model sensitivity of 82.9% and specificity of 76.9%. The *x*-axis denotes 1 − specificity, and the *y*-axis denotes sensitivity.

The model demonstrated acceptable internal discrimination, with an AUC of 0.830 (95% CI, 0.742–0.918). At the optimal threshold (0.724), sensitivity was 82.9%, specificity 76.9%, positive predictive value 91.9%, negative predictive value 58.8%, and accuracy 81.5% (Figure 4). The Hosmer–Lemeshow test (*χ*^2^ = 11.42, *P* *=* 0.179) and Brier score (0.1468) indicated good calibration (Supplementary Figure 2). Bootstrap validation showed an optimism of 0.061 and a bias-corrected C-statistic of 0.768. A nomogram for individualized risk prediction is presented in Supplementary Figure 3.

#### 3.5 Reproducibility of IPN grading

Inter-rater reliability for IPN grading was excellent, with a Cohen's kappa coefficient of 0.887 (95% CI, 0.817–0.887; *P* *<* 0.001) and agreement of 91.67%.

## 4 Discussion

In this study, we investigated the relationship between phase-specific turbulence characteristics derived from VFI and IPN in carotid atherosclerotic plaques. The main finding was that reduced downstream turbulence during end-diastole was significantly associated with IPN positivity. Furthermore, an exploratory model integrating this hemodynamic parameter with clinical and morphological plaque characteristics showed acceptable internal discrimination for identifying plaques with IPN. These findings suggest that phase-specific hemodynamic disturbances may reflect underlying pathological changes related to plaque vulnerability, and that turbulence-derived parameters obtained by VFI could provide additional information for noninvasive assessment of carotid plaque instability.

Tur reflects local flow disturbance by quantifying directional dispersion of blood flow and exhibits dynamic changes across the cardiac cycle. Consistent with previous studies showing that Tur in stenotic carotid segments and downstream regions is higher during systole than during diastole (9), we observed higher Tur during systole than during diastole. Notably, lower downstream end-diastolic Tur was associated with IPN positivity, suggesting that phase-specific flow features may provide additional information on plaque vulnerability. While previous research has emphasized the role of low or oscillatory shear stress in promoting atherosclerosis (18), our findings highlight the potential importance of phase-specific flow characteristics. Experimental evidence has suggested that diastolic flow exposure may activate endothelial homeostasis-related pathways, including Notch signaling (19). Thus, the relatively higher downstream end-diastolic Tur observed in IPN-negative plaques may indicate a preserved phase-specific flow pattern that is potentially relevant to endothelial homeostatic responses.

The association between lower downstream end-diastolic Tur and IPN positivity may also reflect the influence of plaque-related downstream flow organization. Even in healthy arteries, low-level directional dispersion of blood flow can be detected, with reported total average Tur values of 0.46% ± 1.09% in the carotid artery (20). Plaques and stenotic lesions may further reshape downstream flow behavior. Computational fluid dynamics studies have shown that severe stenosis can produce sustained vortices, recirculation, and a high oscillatory shear environment downstream (21, 22). Flow-model studies have also shown that plaque ulceration significantly increases downstream turbulence intensity during both peak systole and diastole (*P* < 0.001) (23). These findings suggest that downstream Tur reflects plaque-related alterations in local flow organization rather than a simple measure of global flow disturbance.

The finding that downstream end-diastolic Tur was significantly lower in IPN-positive plaques, whereas downstream systolic Tur showed no significant difference between groups, may be further explained by the influence of plaque geometry on local hemodynamics. IPN-positive plaques are often associated with greater plaque burden and more complex morphology, including larger plaque size and more irregular surface features (3, 4). These morphological features can reshape the downstream flow field. Computational fluid dynamics studies have shown that increased stenosis length may produce higher flow velocity while allowing translational kinetic energy to dissipate more rapidly, thereby extending the downstream low-Tur jet core region (24). In the present study, the center of the downstream ROI was placed at the middle downstream segment of the plaque. Therefore, in IPN-positive plaques, this ROI may still lie within the low-Tur core region, resulting in a lower measured Tur value. In shorter IPN-negative plaques, the same relative downstream location may already enter the high-Tur shear layer or recirculation region outside the low-Tur core, resulting in a higher measured Tur value.

This interpretation may also explain why the difference was observed during end-diastole rather than systole. During end-diastole, blood velocity and inertial forces are lower, corresponding to a relatively low-Reynolds-number flow environment (25). Under such conditions, jet development distance may be shorter, and the effect of surface irregularity on flow resistance may be attenuated (26). These characteristics could further amplify the influence of plaque length and luminal geometry on the downstream low-Tur core region. Therefore, downstream end-diastolic Tur should be interpreted as an integrative hemodynamic marker that reflects the combined influence of plaque geometry, phase-specific flow conditions, and local downstream flow organization.

While WSS provides valuable information about the hemodynamic environment, it captures only one aspect of flow complexity (27). Recent advances, such as the WSS topological skeleton, have enhanced our understanding of near-wall transport processes (28). Phase-specific Tur may complement these metrics by providing temporal information on flow-direction dispersion. Under physiological conditions, mean WSS and mean Tur are negatively correlated (29), indicating that higher shear stress is generally associated with more orderly flow. However, in pathological states, higher instantaneous WSS has been linked to IPN positivity (30). In the present study, the negative association between downstream end-diastolic Tur and IPN suggests that the relationship between WSS and Tur may be phase-dependent and context-specific. Overall, these findings suggest that the association between downstream end-diastolic Tur and IPN should be regarded as context-specific and hypothesis-generating rather than as evidence of an independent causal hemodynamic mechanism.

From a clinical perspective, VFI-derived Tur is noninvasive, contrast-free, and can be obtained during routine carotid ultrasound assessment. Compared with CEUS or magnetic resonance imaging, VFI is more accessible, widely available, and suitable for routine clinical screening and longitudinal follow-up. Therefore, VFI-derived Tur may provide complementary hemodynamic information for noninvasive IPN assessment.

Several limitations should be acknowledged. This single-center cross-sectional study had a modest sample size and a limited number of IPN-negative plaques, which may increase the risk of model overfitting, while the cross-sectional design limits causal inference. External validation and clinical follow-up were unavailable in the present study.

## 5 Conclusion

In conclusion, reduced downstream end-diastolic Tur was associated with IPN in carotid atherosclerotic plaques. Phase-specific Tur derived from VFI may provide a potential noninvasive hemodynamic marker for IPN assessment.

## Data Availability

The raw data supporting the conclusions of this article will be made available by the authors, without undue reservation.
