# Supplementary material for: Phase-specific turbulence index derived from vector flow imaging for identifying intraplaque neovascularization in carotid plaques
Source: Front Cardiovasc Med. 2026 Jun 17;13:1831573. doi: 10.3389/fcvm.2026.1831573 (PMC13318759; doi:10.3389/fcvm.2026.1831573)
Supplement: Supplementary file 1 [file Table1.docx]

**Supplementary Tab. 1. Model Coefficients**

|  | **Univariable** | | **LASSO (Final Model)** | | |
| --- | --- | --- | --- | --- | --- |
| **Variables** | **OR (95% CI)** | ***p*** | **Coefficient** | **OR** | **Status** |
| DBP, mmHg | 0.96 (0.93–0.99) | **0.01** | **-0.016** | **0.98** | **Selected** |
| Tur-Diastole-Downstream, % | 0.96 (0.93–0.99) | **0.02** | **-0.020** | **0.98** | **Selected** |
| Length, mm | 1.14 (1.05–1.24) | **0.003** | **0.050** | **1.05** | **Selected** |
| Thickness, mm | 2.16 (1.09–4.29) | **0.03** | **0.122** | **1.13** | **Selected** |
| Female | 0.41 (0.16–1.04) | **0.06** | **-0.212** | **0.81** | **Selected** |
| Location (CB) | 0.13 (0.02–1.01) | **0.05** | **-0.254** | **0.78** | **Selected** |
| SBP, mmHg | 0.98 (0.96–1.00) | **0.06** | — | — | Not selected |
| Tur-Downstream, % | 0.96 (0.92–1.00) | **0.07** | — | — | Not selected |
| HbA1c, % | 1.36 (0.92–2.00) | 0.12 | — | — | Not selected |
| Age, years | 1.03 (0.99–1.08) | 0.15 | — | — | Not selected |
| RI | 13.25 (0.25–706.40) | 0.20 | — | — | Not selected |
| Diabetes | 1.95 (0.66–5.75) | 0.23 | — | — | Not selected |
| Tur-Diastole-Upstream, % | 0.91 (0.77–1.06) | 0.23 | — | — | Not selected |
| Tur-Systole-Downstream, % | 0.99 (0.97–1.01) | 0.23 | — | — | Not selected |
| Tur-Diastole-Maximum thickness, % | 0.98 (0.95–1.02) | 0.34 | — | — | Not selected |
| Location (ICA) | 0.38 (0.04–3.67) | 0.40 | — | — | Not selected |
| Hypertension | 0.69 (0.28–1.70) | 0.42 | — | — | Not selected |
| Smoke exposure | 0.70 (0.29–1.70) | 0.43 | — | — | Not selected |
| Tur-Upstream, % | 0.98 (0.93–1.03) | 0.48 | — | — | Not selected |
| Tur-Systole-Maximum thickness, % | 0.99 (0.98–1.01) | 0.51 | — | — | Not selected |
| Tur-Maximum thickness, % | 0.99 (0.95–1.03) | 0.64 | — | — | Not selected |
| TG, mmol/L | 1.02 (0.94–1.10) | 0.68 | — | — | Not selected |
| PSV, cm/s | 1.00 (0.99–1.01) | 0.68 | — | — | Not selected |
| BMI | 0.98 (0.90–1.07) | 0.71 | — | — | Not selected |
| HCY, mmol/L | 1.01 (0.97–1.04) | 0.72 | — | — | Not selected |
| HDL/CHOL | 0.99 (0.94–1.05) | 0.73 | — | — | Not selected |
| Tur-Systole-Upstream% | 1.00 (0.98–1.03) | 0.76 | — | — | Not selected |
| TC, mmol/L | 1.06 (0.69–1.62) | 0.79 | — | — | Not selected |
| LDL, mmol/L | 1.07 (0.58–1.97) | 0.82 | — | — | Not selected |
| EDV, cm/s | 1.00 (0.99–1.02) | 0.88 | — | — | Not selected |
| HDL, mmol/L | 0.96 (0.19–4.87) | 0.96 | — | — | Not selected |

Abbreviations: Tur, turbulence index; BMI, Body mass index; CB, carotid bifurcation; HbA1c, hemoglobin A1c; HCY, homocysteine; HDL, high-density lipoprotein; ICA, internal carotid artery; LDL, low-density lipoprotein; HDL/CHOL, high-density lipoprotein/cholesterol PSV, peak systolic velocity; RI, resistance index; TC, total cholesterol; TG, triglycerides. OR, odds ratio; CI, confidence interval.

LASSO model selected using lambda = lambda.1se (0.046860).

Coefficients and ORs are presented for selected variables; '—' indicates variable not selected.

Boldface entries indicate statistical significance (*p < 0.1*) and selected status.
