# Supplementary material for: Phase-specific turbulence index derived from vector flow imaging for identifying intraplaque neovascularization in carotid plaques
Source: Front Cardiovasc Med. 2026 Jun 17;13:1831573. doi: 10.3389/fcvm.2026.1831573 (PMC13318759; doi:10.3389/fcvm.2026.1831573)
Supplement: Supplementary file 2 [file Datasheet1.docx]

# **Supplementary Figures legends**

## **Supplementary Fig 1.** LASSO λ selection and the coefficient path map of the LASSO regression model.

A shows the validation results for LASSO λ selection, and B presents the coefficient path map of the LASSO regression model, with λ.1se = 0.0469. LASSO regression selected 6 independent predictors from 8 candidate variables: diastolic blood pressure (DBP), Tur-Diastole-Downstream, Length, Thickness, Female, and carotid bifurcation.

## **Supplementary Fig 2.** Final model calibration curve.

Hosmer-Lemeshow test p = 0.179, Brier score 0.1468, indicating good model calibration.

## **Supplementary Fig 3.** Intraplaque neovascularization (IPN) positivity bar chart.

Sex1 and Sex2 denote males and females, respectively; Location1, Location2, and Location3 denote CCA, CB, and ICA, respectively. DBP denotes diastolic blood pressure.
