# Supplementary figures and images for: Phase-specific turbulence index derived from vector flow imaging for identifying intraplaque neovascularization in carotid plaques
Source: Front Cardiovasc Med. 2026 Jun 17;13:1831573. doi: 10.3389/fcvm.2026.1831573 (PMC13318759; doi:10.3389/fcvm.2026.1831573)

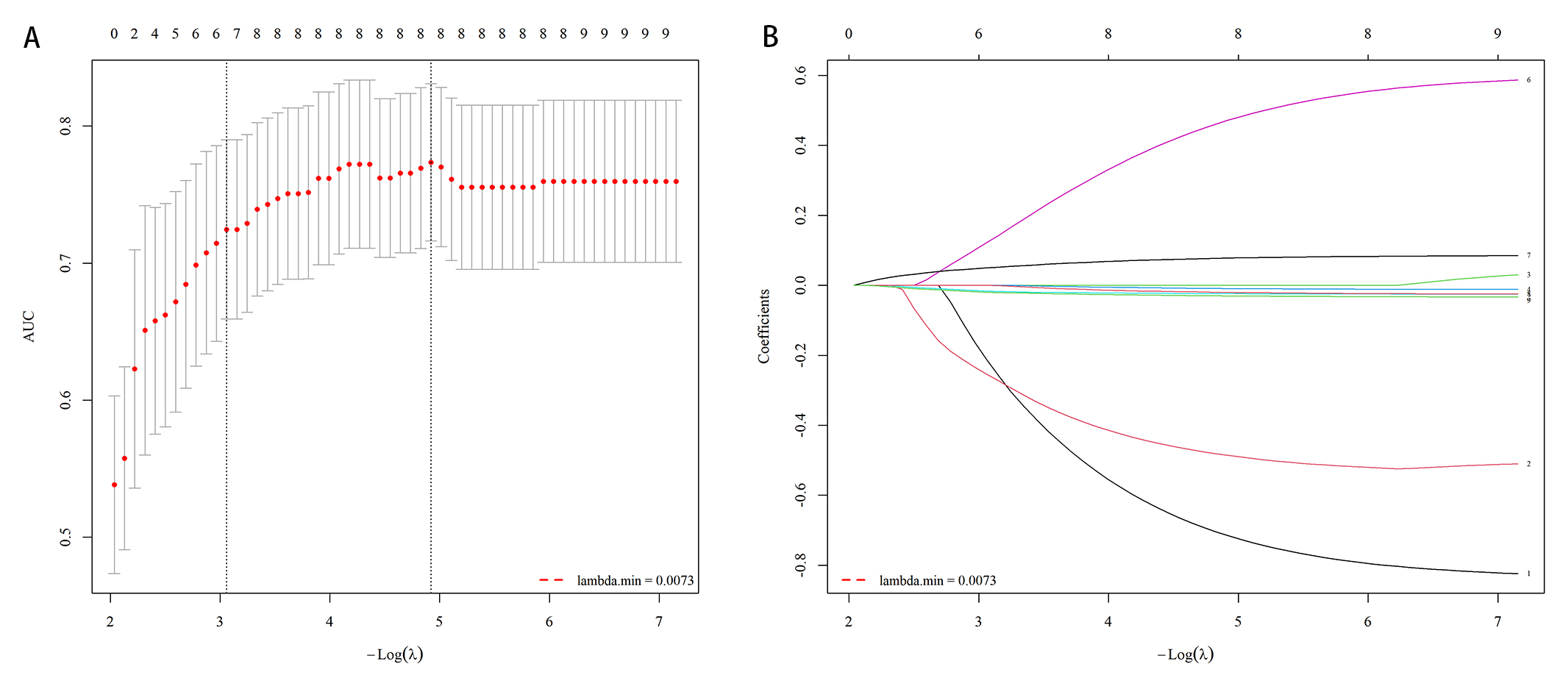

Supplement: Supplementary file 3 [file Image1.jpeg]

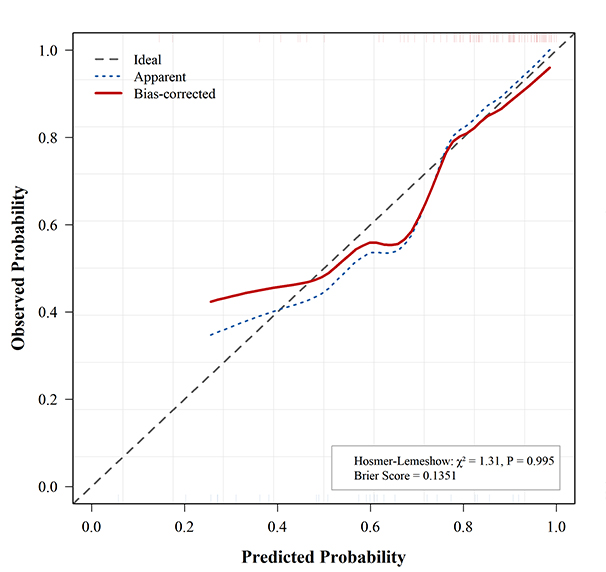

Supplement: Supplementary file 4 [file Image2.jpeg]

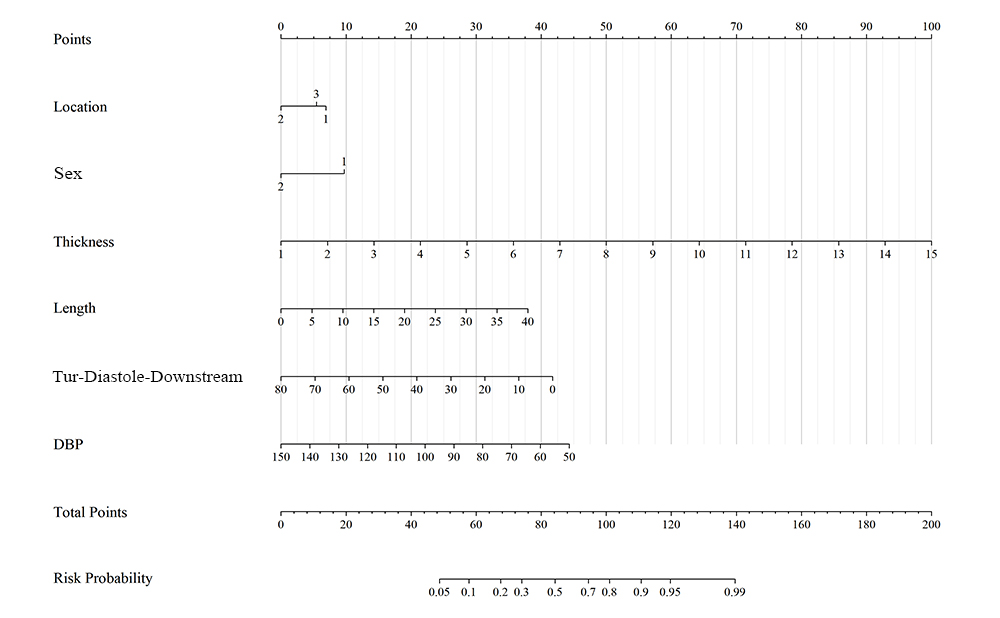

Supplement: Supplementary file 5 [file Image3.jpeg]
